# Autonomous navigation in unstructured outdoor environments using semantic segmentation guided reinforcement learning

**DOI:** 10.1038/s41598-026-36022-2

**Published:** 2026-01-20

**Authors:** Ahmed Tibermacine, Imad Eddine Tibermacine, Djouher Akrour, Abdelaziz Rabehi, Mustapha Habib

**Affiliations:** 1 LESIA Laboratory, Department of Computer Science, University of Biskra, Biskra, Algeria; 2 Department of Computer, Automation and Management Engineering, Sapienza University of Rome, Rome, Italy; 3 Laboratory of Telecommunication and Smart Systems (LTSS), Faculty of Science and Technology, University of Djelfa, 17000 Djelfa, Algeria; 4 Division of Building Technology and Design Department of Civil and Architectural Engineering, KTH Royal Institute of Technology, Stockholm, Sweden

**Keywords:** Engineering, Mathematics and computing

## Abstract

Robust autonomous navigation in dense, unstructured environments such as forests presents a longstanding challenge in robotics due to complex terrain geometry, dynamic occlusions, and unreliable global positioning signals. This paper proposes a hybrid perception-and-control framework that integrates deep semantic segmentation with reinforcement learning to enable intelligent, vision-driven navigation in visually cluttered forest trails. The system combines Mask R-CNN for pixel-level trail segmentation with a Soft Actor-Critic (SAC) agent that learns adaptive navigation policies under continuous action spaces. A Pure Pursuit controller translates visual predictions into smooth motor commands, ensuring path adherence and stability. The model is trained and evaluated in a high-fidelity forest simulation environment featuring natural obstacles, variable lighting, and randomized trail geometries. Extensive experiments demonstrate that our approach achieves a high trail-following success rate (86.7%), low collision frequency, and precise path tracking in challenging navigation scenarios. Comparative and ablation studies further highlight the synergy between learning-based perception and control. The proposed framework offers a scalable and modular solution for deploying autonomous robots in natural terrains without relying on GPS or prior maps, paving the way for applications in environmental monitoring and field robotics.

## Introduction

Autonomous robot navigation in unstructured outdoor environments remains one of the most challenging and important problems in the field of robotics. Forests, in particular, represent highly complex, dynamic, and visually cluttered terrains where traditional navigation methods struggle due to factors such as uneven surfaces, dense vegetation, occlusions, and poor GPS reliability. Robust autonomous navigation in these environments holds significant potential for real-world applications, including environmental monitoring, forest management^1^, search and rescue operations^2^, and precision agriculture^3,4^. For a robot to navigate these domains effectively, it must possess both perceptual intelligence to interpret natural scenes and decision-making intelligence to adapt its behavior in real time to unpredictable surroundings.

Conventional approaches to outdoor robot navigation often rely on GPS-based localization and handcrafted features for obstacle detection and path planning. However, these methods are brittle in dense natural environments, where GPS signals can be severely degraded and visual conditions are highly variable^5,6^. Furthermore, purely rule-based navigation systems are typically unable to generalize across diverse terrain types or recover gracefully from ambiguous or occluded situations. Recent progress in deep learning has significantly advanced perception and decision-making capabilities in autonomous systems. In computer vision, deep convolutional architectures have demonstrated remarkable efficiency in learning complex spatial representations^7,8^. More recently, semantic segmentation networks have further improved robustness and precision in visual understanding^9,10^. Similarly, reinforcement learning approaches^11–13^ and hybrid perception–learning frameworks^14,15^ have opened promising avenues toward more intelligent and adaptable autonomous navigation.

Despite these advances, several key research gaps persist. First, most existing vision-based forest navigation systems rely on image-level classification or bounding-box detection, which lack the pixel-level spatial precision required to navigate narrow and winding trails. Second, while reinforcement learning has achieved notable success in structured or indoor domains, its application to visually complex, partially observable outdoor environments, including forests, remains underexplored, especially within continuous control formulations. Third, few studies integrate semantic perception, adaptive learning, and classical control into a unified real-time navigation pipeline that is simultaneously interpretable, robust, and deployable on resource-constrained robotic platforms.

To address these gaps, this paper presents a novel hybrid autonomous navigation system that integrates deep visual perception with entropy-regularized reinforcement learning for robust trail-following in dense forest environments.Our primary contributions are

- *A tailored Mask R-CNN-based perception module* for pixel-accurate trail segmentation in visually cluttered and occluded forest scenes. The module is optimized for real-time execution on embedded hardware and generalizes across varying illumination, vegetation density, and trail geometry.
- *The adaptation of the SAC algorithm* to continuous-control navigation in partially observable, unstructured outdoor terrain. We design a reward function that encourages trail adherence, collision avoidance, and efficient goal-reaching while maintaining exploratory behavior through entropy regularization.
- *A modular and interpretable navigation architecture* that seamlessly integrates semantic segmentation (Mask R-CNN), adaptive decision-making (SAC), and geometric path tracking (Pure Pursuit) into a real-time pipeline. This hybrid design balances the flexibility of learning-based methods with the stability and transparency of classical control.
- *Extensive experimental validation* in high-fidelity simulated forest environments featuring diverse navigational challenges, including narrow trails, rugged terrain, elevation changes, and trail bifurcations. The proposed system achieves a high success rate of 86.7%, low collision frequency, and precise path tracking without reliance on GPS or prior maps, outperforming a range of contemporary methods across multiple perceptual modalities.

The principal innovation of our system resides in its unified integration of three complementary components: pixel-level semantic perception, sample-efficient reinforcement learning under uncertainty, and reliable geometric control. This integration is implemented within a modular architecture that enables real-time execution and rigorous evaluation. In contrast to end-to-end black-box models, our methodology maintains interpretability across all functional modules, specifically perception, decision-making, and control. This design choice enhances system transparency and supports practical benefits such as improved debugging, adaptation to new environmental conditions, and transferability across diverse application domains.

In the remainder of this paper, Section 2 reviews related work in outdoor navigation, semantic perception, and reinforcement learning. Section 3 details the proposed architecture, including the perception, decision, and control modules. Section 4 describes the experimental setup, including simulation environments, dataset, training parameters, and evaluation metrics. Section 5 presents quantitative and qualitative results, along with ablation and comparative studies. Section 6 discusses the findings, limitations, and broader implications. Finally, Section 7 concludes the paper and outlines future research directions.

## Related work

Autonomous navigation in unstructured outdoor environments has been extensively investigated in robotics and AI communities. Classical methods, such as those that rely on GPS-based localization^16^, Simultaneous Localization and Mapping (SLAM)^17^, or LiDAR-based occupancy grids^18^, are well-established for structured and semi-structured terrains. However, these approaches exhibit performance degradation in cluttered natural environments like forests, where GPS signals are weak, visual scenes are ambiguous, and the geometry of terrain is complex^19,20^. Vision-based alternatives have been proposed to address these shortcomings, enabling navigation in environments without reliance on GPS or high-precision maps^21,22^.

Early visual navigation systems often relied on handcrafted features such as color histograms, HOG, or edge-based filters to detect trails and free space. Although computationally efficient, these methods are highly sensitive to lighting variations, seasonal changes, and occlusions. Wang et al.^23^ presented an object-based visual navigation system that enhances the reliability of mobile robots by utilizing environmental objects for localization and path planning in dynamic environments. In a separate study, Niu et al.^24^ introduced a surprisingly simple yet effective visual navigation method tailored to forest environments, demonstrating that minimalistic approaches can still enable robust robotic navigation in complex natural settings. The introduction of machine learning and deep learning marked a turning point, leading to the development of more robust systems. Studies such as^21,25^ showed that these techniques can successfully identify forest trails from ground images, offering significant improvements in robustness compared to traditional methods. However, many of these early models focused on image-level classification or bounding box detection^23,26,27^, which limits their spatial precision and makes them less suitable for real-time navigation in narrow or winding trails^28^.

Instance and semantic segmentation architectures such as Mask R-CNN^29^, DeepLab^30^, and PSPNet^31^ have demonstrated exceptional performance in pixel-level scene understanding across various domains, including autonomous driving^32^, aerial surveying^33^, and robotics^34^. In forested environments, semantic segmentation has been employed to detect vegetation, obstacles, or drivable paths. Umar et al.^35^ applied this approach to identify walkable regions in forest scenes captured by drones. Valada et al.^36^ developed an ensemble model aimed at terrain classification in agricultural fields. Despite these advances, most existing methods are either tailored to specific terrain types or struggle with real-time performance due to high computational demands. Our work builds on the strengths of Mask R-CNN while introducing specific adaptations for real-time trail segmentation in visually ambiguous natural terrains, thereby improving navigability for ground robots in forest environments.

Reinforcement learning (RL) has rapidly gained prominence for end-to-end autonomous control, particularly in robotic manipulation, locomotion, and navigation. Early successes like DQN^37^ and A3C^38^ in Atari and simulated environments led to more sophisticated algorithms tailored for continuous control, such as DDPG^39^, PPO^40^, and TD3^41^. SAC^42^ has emerged as a strong candidate for robotic navigation due to its sample efficiency and ability to avoid premature policy convergence. Applications of SAC have been reported in robotic arm control^43^, drone flight^44^, and indoor mobile navigation^45^. However, relatively few studies have applied SAC in natural outdoor settings where perception uncertainty, sensor noise, and environmental variability are significantly higher.

Some recent efforts have explored integrating semantic perception with reinforcement learning to form hybrid navigation pipelines. Santos et al.^46^ combined semantic segmentation with PPO for indoor room-to-room navigation. Faust et al.^47^ proposed PRM-RL, which uses a roadmap planner for high-level goals and RL for local control in complex terrains. Chen et al.^48^ integrated CNN-based visual perception with RL for mapless navigation in crowded human environments. These hybrid models balance interpretability and adaptability, but are typically evaluated in structured or simulated indoor domains. Our contribution advances this line of research by demonstrating that pixel-accurate semantic segmentation, when coupled with entropy-regularized reinforcement learning, enables robust long-range navigation in visually complex and unstructured outdoor forest scenarios.

Moreover, control-oriented components such as Pure Pursuit or Dynamic Window Approaches (DWA) are often employed to track high-level path plans^49^. Integrating these classical geometric methods with learning-based perception has been shown to enhance stability and interpretability, particularly in safety-critical contexts. Dhanya et al.^50^ applied semantic segmentation alongside Pure Pursuit to guide agricultural field robots, and Pierre et al.^51^ incorporated a similar strategy for navigation in orchards using deep perception. Our system preserves this modular structure by combining learned perception and adaptive decision-making with a reliable geometric controller, enabling real-time performance in robotic applications.

The existing literature demonstrates substantial advances in both semantic perception and reinforcement learning for robotic navigation. Despite this progress, there remains a clear gap in combining these approaches into a modular real-time system tailored to dense forest environments. Our work addresses this challenge by integrating Mask R-CNN, SAC, and Pure Pursuit into a cohesive navigation pipeline, providing a scalable and robust framework for autonomous navigation in unstructured natural settings.

## Proposed Model

### System Architecture Overview

**Fig. 1:**
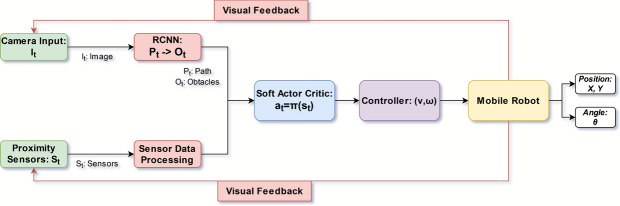
Overview of the proposed hybrid navigation architecture. The system integrates a Mask R-CNN-based visual segmentation module and SAC reinforcement learning policy to guide robot control based on sensor fusion.

The proposed autonomous navigation framework is structured as a multi-layered, modular architecture designed for real-time, perception-driven decision-making in unstructured forest environments. It consists of three tightly coupled subsystems: (i) a deep learning-based visual perception module, (ii) a policy learning-based reinforcement learning navigation module, and (iii) a low-level control execution system. These subsystems are orchestrated in a closed feedback loop to ensure continuous environment understanding, optimal path selection, and safe trajectory tracking (see Fig. 1).

#### Visual perception module

At each timestep *t*, the robot captures an RGB frame $I_t \in {\mathbb {R}}^{H \times W \times 3}$ using an onboard monocular camera mounted in the forward-facing direction. This image is fed into a pretrained Mask R-CNN segmentation network, which produces a binary trail segmentation mask defined by:

1 $$\begin{aligned} M_t(x, y) = {\left\{ \begin{array}{ll} 1 & \text {if pixel } (x, y) \text { belongs to a navigable trail}, \\ 0 & \text {otherwise}. \end{array}\right. } \end{aligned}$$

Based on this binary mask, the set of trail pixels is extracted as

2 $$\begin{aligned} {\mathscr {T}}_t = \left\{ (x_i, y_i) \in {\mathbb {R}}^2 \mid M_t(x_i, y_i) = 1 \right\} . \end{aligned}$$

To obtain a compact and navigable representation of the segmented trail, a parametric centerline curve $\tau _t(s): [0, L] \rightarrow {\mathbb {R}}^2$ is fitted to ${\mathscr {T}}_t$ using spline interpolation, where *s* denotes the arc length parameter and *L* is the estimated length of the visible trail. Subsequently, this centerline is used to compute the geometric features of the path, such as the curvature, heading, and lateral offset.

The robot’s complete state vector $s_t \in {\mathscr {S}}$ is then constructed by fusing geometric features with proprioceptive information, and is expressed as:

3 $$\begin{aligned} s_t = \left[ x_t, y_t, \theta _t, \Delta \theta _t, \min _{s \in [0, L]} \left\| (x_t, y_t) - \tau _t(s) \right\| , {\textbf{p}}_t \right] , \end{aligned}$$

where $(x_t, y_t, \theta _t)$ denotes the robot’s pose in the environment, $\Delta \theta _t$ is the angular deviation between the robot’s heading and the local trail tangent at the closest point on $\tau _t$, and ${\textbf{p}}_t$ represents the robot’s vector of proximity sensor readings.

#### Reinforcement learning module

The high-level decision-making of the robot is governed by an SAC agent, a model-free, off-policy reinforcement learning algorithm optimized for continuous action spaces. Given a state $s_t \in {\mathscr {S}}$, the stochastic policy network $\pi _\phi (a_t | s_t)$, parameterized by $\phi$, outputs a distribution over actions $a_t = (\nu _t, \omega _t)$, representing the linear and angular velocities of the robot.

SAC optimizes a stochastic policy by maximizing the expected **entropy-augmented return**, which encourages both high rewards and high policy entropy (i.e., exploration):

4 $$\begin{aligned} J(\pi ) = {\mathbb {E}}_{\tau \sim \rho _\pi } \left[ \sum _{t=0}^\infty \gamma ^t \left( r(s_t, a_t) + \alpha {\mathscr {H}}(\pi (\cdot | s_t)) \right) \right] , \end{aligned}$$

where $\gamma \in [0,1]$ is the discount factor and $\alpha > 0$ is the temperature coefficient that controls the importance of the entropy term:

5 $$\begin{aligned} {\mathscr {H}}(\pi (\cdot | s_t)) = -{\mathbb {E}}_{a_t \sim \pi } \left[ \log \pi (a_t | s_t) \right] . \end{aligned}$$

To estimate the action-value function, SAC employs two soft Q-networks $Q_{\theta _1}$ and $Q_{\theta _2}$ with parameters $\theta _1$ and $\theta _2$, respectively. These networks are trained to minimize the soft Bellman residual:

6 $$\begin{aligned} J_Q(\theta _i) = {\mathbb {E}}_{(s_t, a_t, r_t, s_{t+1}) \sim {\mathscr {D}}} \left[ \left( Q_{\theta _i}(s_t, a_t) - \hat{y}_t \right) ^2 \right] , \end{aligned}$$

for $i \in \{1,2\}$, where the soft target value $\hat{y}_t$ is calculated using a target policy and target Q-networks $(\bar{\theta }_1, \bar{\theta }_2)$:

7 $$\begin{aligned} \hat{y}_t = r_t + \gamma \left( \min _{i=1,2} Q_{\bar{\theta }_i}(s_{t+1}, a_{t+1}) - \alpha \log \pi _\phi (a_{t+1} | s_{t+1}) \right) , \end{aligned}$$

with $a_{t+1} \sim \pi _\phi (\cdot | s_{t+1})$. The use of the minimum of the two Q-functions helps mitigate the overestimation bias commonly encountered in Q-learning methods.

The policy network $\pi _\phi$ is updated simultaneously to minimize the expected KL divergence between the policy and the exponential of the Q-value, effectively encouraging actions with higher expected returns and higher entropy.

#### Low-level control module

To ensure smooth and stable path following, the system incorporates a geometric controller based on the Pure Pursuit algorithm. At each timestep *t*, the controller selects a goal point ${\textbf{g}}_t = (x_g, y_g)$ along the centerline of the fabricated trail $\tau _t(s)$, located at a fixed lookahead distance $L_d$ from the robot’s current position. Assuming a differential-drive robot model, the required curvature $\kappa _t$ to reach ${\textbf{g}}_t$ is given by:

8 $$\begin{aligned} \kappa _t = \frac{2 y_g}{L_d^2}, \end{aligned}$$

where $y_g$ is the lateral offset of the target point in the robot’s local coordinate frame. The curvature $\kappa _t$ is then mapped to linear and angular velocities using a control function:

9 $$\begin{aligned} (\nu _t^{\text {PP}}, \omega _t^{\text {PP}}) = f(\kappa _t), \end{aligned}$$

where $f(\cdot )$ is a control law that maintains a constant forward speed $\nu _t$ and adjusts the turning rate $\omega _t$ based on the desired curvature.

To enhance flexibility and robustness, the system supports an optional blending of SAC-generated actions with those from the Pure Pursuit controller. The final action is computed as a convex combination:

10 $$\begin{aligned} a_t^{\text {final}} = \lambda a_t^{\text {SAC}} + (1 - \lambda ) a_t^{\text {PP}}, \quad \lambda \in [0, 1], \end{aligned}$$

where $a_t^{\text {SAC}}$ and $a_t^{\text {PP}}$ denote the actions (linear and angular velocities) of the SAC agent and the Pure Pursuit controller, respectively. The weight of blending $\lambda$ can be tuned statically or dynamically based on contextual information such as the curvature of the trail, the complexity of the terrain, or the uncertainty of the learned policy.

### Visual perception with mask R-CNN

The visual perception module serves as the entry point of the autonomous navigation pipeline, providing essential environmental understanding for subsequent decision-making and control. segmentation is designed to deliver timely and sufficiently accurate geometric cues of the navigable trail from monocular RGB imagery, within the real-time and computational constraints of mobile robots operating in complex forest environments.

From a system-level perspective, the use of a lightweight and reliable segmentation approach ensures real-time execution and seamless integration with the control pipeline. The module processes incoming frames at a rate compatible with the navigation loop, continuously providing updated trail geometry for policy evaluation and control synthesis. This trade-off prioritizes responsiveness and robustness over marginal improvements in pixel-level accuracy, which offer limited benefits for practical robot navigation.

**Fig. 2:**
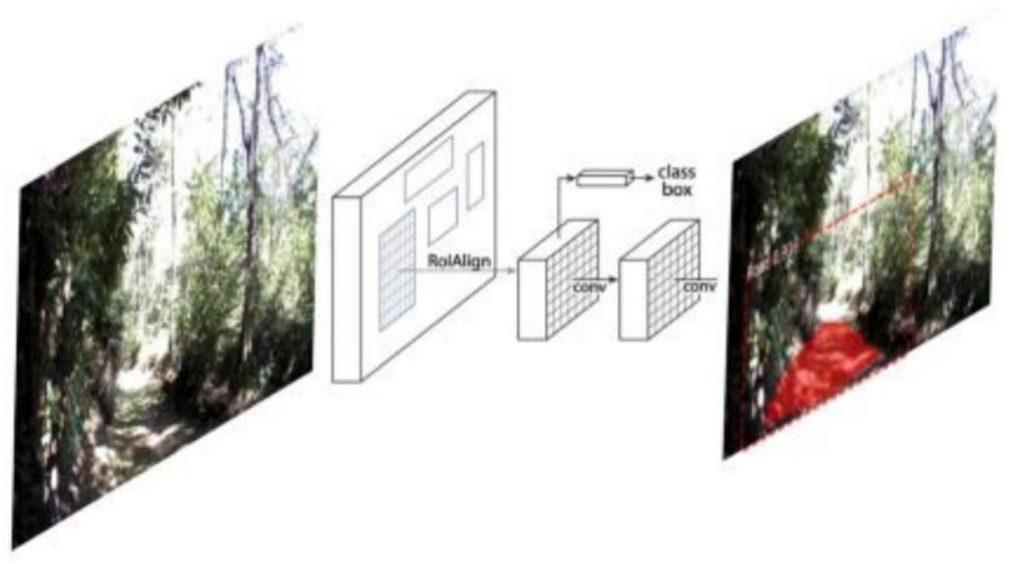
Mask R-CNN-based visual perception pipeline. The RGB image is passed through a ResNet-50 + FPN backbone. The Region Proposal Network (RPN) generates candidate regions, which are refined via RoIAlign and processed through classification and segmentation heads. The output is a per-pixel binary mask of the navigable trail, used downstream for decision-making and control.

To identify navigable trails, we employ a Mask R-CNN-based segmentation network that converts each RGB frame $I_t \in {\mathbb {R}}^{H \times W \times 3}$ into a binary mask $M_t \in \{0,1\}^{H \times W}$, where trail pixels are marked as 1. The network, built on a ResNet-50 backbone with a Feature Pyramid Network (FPN), extracts multiscale features processed through a Region Proposal Network (RPN), RoIAlign, and parallel classification and segmentation heads. The complete inference process, from the raw image to the trail segmentation output, is illustrated in Fig. 2.

The detected trail masks are aggregated, denoised using morphological operations, and converted into a skeletonized centerline ${\mathscr {P}}_t$, which is fitted with a smooth parametric curve $\tau _t(s)$. This curve serves as a shared input to the decision-making module, where it is encoded into the robot’s state vector for policy learning, and to the motion control module, which uses the curve to generate curvature-based path-following commands. By converting raw visual data into structured geometric information, the perception module effectively bridges the high-dimensional visual input space with the lower-dimensional, decision-ready state representations needed by the SAC policy and the Pure Pursuit controller.

### Navigation strategy with SAC

Autonomous navigation in unstructured and partially observable environments, such as dense forests, requires a control strategy that is both adaptive and robust to dynamic sensory uncertainty. To this end, the proposed system employs the SAC algorithm as its core decision-making module. SAC is an off-policy, actor-critic reinforcement learning method optimized for continuous control, combining stable value approximation with the exploratory benefits of maximum entropy reinforcement learning. This enables the agent to cope with variable trail geometries, occlusions, and sensory ambiguity in a principled and sample-efficient manner.

The navigation task is modeled as a Markov Decision Process (MDP) defined by the tuple:

$${\mathscr {M}} = ({\mathscr {S}}, {\mathscr {A}}, {\mathscr {P}}, {\mathscr {R}}, \gamma ),$$

where ${\mathscr {S}} \subseteq {\mathbb {R}}^n$ is the continuous state space, ${\mathscr {A}} \subseteq {\mathbb {R}}^m$ the action space (comprising linear and angular velocities), ${\mathscr {P}}$ the environment’s transition dynamics, ${\mathscr {R}}$ the reward function, and $\gamma \in (0, 1]$ the discount factor.

At each time step *t*, the agent observes a state $s_t \in {\mathscr {S}}$, which includes the robot’s current pose, heading deviation from the trail centerline, proximity sensor readings, and curvature estimates from the segmented trail. Based on this observation, the stochastic policy $\pi _\phi (a_t | s_t)$, parameterized by $\phi$, generates a continuous action $a_t = (\nu _t, \omega _t)$, where $\nu _t$ and $\omega _t$ are the linear and angular velocities, respectively.

The SAC objective is to learn a policy that maximizes the expected entropy-regularized cumulative return:

$$J(\pi ) = \sum _{t=0}^{\infty } {\mathbb {E}}_{(s_t, a_t) \sim \rho _\pi } \left[ r(s_t, a_t) + \alpha {\mathscr {H}}(\pi (\cdot | s_t)) \right] ,$$

where the entropy term is defined as:

$${\mathscr {H}}(\pi (\cdot | s_t)) = -{\mathbb {E}}_{a_t \sim \pi } \left[ \log \pi (a_t | s_t) \right] ,$$

and the temperature coefficient $\alpha > 0$ modulates the balance between exploration and exploitation.

SAC maintains two soft Q-value functions $Q_{\theta _1}(s, a)$ and $Q_{\theta _2}(s, a)$, along with a stochastic policy network $\pi _\phi (a | s)$. These networks are optimized using the following objectives.

*Critic update (soft Bellman residual):*

$${\mathscr {L}}_Q(\theta _i) = {\mathbb {E}}_{(s_t, a_t, r_t, s_{t+1}) \sim {\mathscr {D}}} \left[ \left( Q_{\theta _i}(s_t, a_t) - \hat{y}_t \right) ^2 \right] ,$$

with the soft target defined as:

$$\hat{y}_t = r_t + \gamma \left( \min _{j=1,2} Q_{\theta _j}(s_{t+1}, a_{t+1}) - \alpha \log \pi _\phi (a_{t+1} | s_{t+1}) \right) .$$

*Policy update:*

$${\mathscr {L}}_\pi (\phi ) = {\mathbb {E}}_{s_t \sim {\mathscr {D}}} \left[ {\mathbb {E}}_{a_t \sim \pi _\phi } \left[ \alpha \log \pi _\phi (a_t | s_t) - Q_{\theta _1}(s_t, a_t) \right] \right] .$$

The reward function is designed to encourage progression along the segmented trail while penalizing deviations, collisions, or stagnation. Specifically, it assigns positive rewards for forward movement on the trail, negative rewards for collisions or leaving the trail bounds, and higher weights for reaching navigation goals. This reward shaping enables the policy to learn stable, safe, and efficient trail-following behaviors.

The SAC agent interfaces directly with the visual perception module by consuming geometric descriptors derived from the segmented trail. Its output actions can be applied directly to the robot or blended with control actions from the Pure Pursuit controller, providing both the adaptability of learning and the stability of classical control.

### Control system

To ensure smooth and stable motion execution, the proposed navigation architecture incorporates a Pure Pursuit Controller as a low-level path tracking module. While the high-level SAC agent governs strategic action selection, the Pure Pursuit controller is responsible for geometric tracking of the trail path extracted by the perception module. Its inclusion introduces a deterministic control layer that complements the learned policy, particularly in scenarios involving tight trail curvature, sensory noise, or limited generalization capacity of the reinforcement learning agent.

The Pure Pursuit algorithm is a nonlinear geometric controller that translates a desired trajectory, expressed as a parametric curve, into continuous control commands based on the robot’s current pose. Let the robot’s pose at time *t* be denoted as ${\textbf{x}}_t = (x_t, y_t, \theta _t)$, where $(x_t, y_t)$ is the 2D position and $\theta _t$ is the heading angle. The trail path, computed from Mask R-CNN segmentation and centerline estimation, is represented as a continuous curve $\tau _t(s): [0, 1] \rightarrow {\mathbb {R}}^2$.

The controller selects a lookahead point ${\textbf{g}}_t = \tau _t(s^*)$ along the path, such that the Euclidean distance $\Vert {\textbf{g}}_t - (x_t, y_t) \Vert = L_d$, where $L_d$ is the lookahead distance. The required curvature $\kappa _t$ of the circular arc connecting the robot to this point is given by:

11 $$\begin{aligned} \kappa _t = \frac{2 y_g}{L_d^2}, \end{aligned}$$

where $y_g$ is the lateral displacement of ${\textbf{g}}_t$ in the robot’s local coordinate frame. The desired angular velocity is computed as

12 $$\begin{aligned} \omega _t = \nu _t \cdot \kappa _t, \end{aligned}$$

where $\nu _t$ is the robot’s linear velocity. In practice, $\nu _t$ can be set as a constant or dynamically modulated based on the confidence of the trail segmentation or the proximity of the obstacle. The resulting control command is expressed as a vector:

$$a_t^{\text {PP}} = (\nu _t, \omega _t) \in {\mathbb {R}}^2.$$

This low-level controller can operate in two modes:

- *Direct execution:* Used standalone for reactive path tracking in clearly segmented or low-complexity regions.
- *Policy fusion:* Combined with the SAC agent’s output $a_t^{\text {SAC}}$ to improve control robustness:
  13 $$\begin{aligned} a_t^{\text {final}} = \lambda \cdot a_t^{\text {SAC}} + (1 - \lambda ) \cdot a_t^{\text {PP}}, \end{aligned}$$
  where $\lambda \in [0, 1]$ is a tunable fusion coefficient that balances classical and learned control.

The Pure Pursuit controller provides effective short-horizon stability and trajectory smoothing, which compensates for potentially noisy or suboptimal decisions from the SAC agent. Its ability to correct minor deviations in real time reduces oscillations and abrupt steering behaviors, ultimately improving navigation safety and efficiency.

To maintain responsiveness in real-world deployment, the controller operates at a higher frequency (e.g. 10–20 Hz) than the high-level SAC policy (e.g. 5 Hz), enabling rapid corrections between policy updates. This frequency separation ensures a tightly closed control loop, particularly valuable in rough or dynamically changing terrain.

The Pure Pursuit controller acts as a critical intermediary between visual perception and physical actuation. Translates high-level geometric paths into smooth, low-level motor commands, reinforcing the interpretability, stability, and real-time capability of the overall navigation system.

## Experimental Setup

This section describes the experimental framework used to validate the proposed navigation system, including the simulation environment, robot platform and hardware configuration, dataset preparation, network architectures, reward function design, and evaluation metrics.

### Simulation Environment

To evaluate the performance and generalization of the proposed hybrid navigation model, we constructed three simulated dense forest environments: Map A, Map B, and Map C. Each environment was designed to reflect different navigational challenges. These environments were implemented in a high-fidelity simulator that includes realistic vegetation, terrain, lighting, and obstacles to closely mimic real-world forest conditions. The variation across these maps enables a comprehensive assessment of both visual segmentation and decision-making capabilities.

#### Map A: Narrow trail with dense vegetation

This map simulates a forest with dense vegetation and narrow, winding trails. Although the terrain is relatively flat, it is visually cluttered with thick undergrowth, low-hanging branches, and frequent occlusions. Trail widths range from 0.6 to 1.2 meters. Map A primarily serves to evaluate the segmentation accuracy of the Mask R-CNN model under conditions of poor visibility and high environmental noise.

#### Map B: Rugged terrain with elevation changes

Map B features uneven terrain with elevation changes, sharp inclines, and irregular paths. Vegetation is less dense than in Map A, but the trail is frequently interrupted by large obstacles such as fallen logs and tree roots. This setting is used to test the SAC agent’s ability to plan and adapt movement on challenging, uneven ground while avoiding static obstacles.

#### Map C: Trail bifurcations and distractors

This environment focuses on evaluating decision-making in ambiguous and misleading scenarios. It contains multiple intersecting trails, including false paths and dead ends. Vegetation is moderately dense, and the trail geometry includes bifurcations and forks. Map C challenges the SAC agent to identify the correct trail based on incomplete or noisy visual input.

All three environments were generated procedurally with randomized obstacle placement and lighting conditions to capture the variability found in real forest settings. The camera viewpoint, field of view, and image resolution were kept consistent in all simulations to ensure a uniform perception input. Each map was designed to introduce a specific type of challenge: perceptual occlusion, topographic complexity, or semantic ambiguity. This setup supports a thorough and balanced evaluation of the proposed hybrid navigation system in a range of forest navigation scenarios.

### Robot platform and hardware configuration

**Fig. 3:**
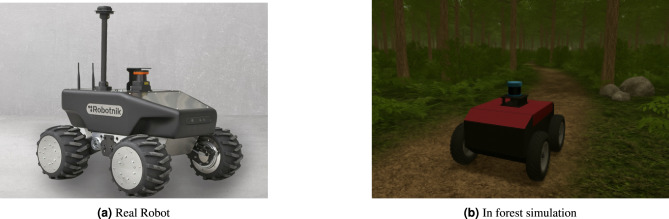
Views of the robot used in our experiments: (**a**) The real robot and (**b**) Forest simulation environment.

The robot used in this study is a compact four-wheeled differential drive platform built for unstructured environments such as dense forests. Its chassis measures 0.5 $\times$ 0.4 $\times$ 0.3 meters, made from lightweight aluminum with a polycarbonate shell. Independent motor control on each side allows for precise turning and pivoting, essential for narrow trails. High-traction rubber tires and 8–10 cm ground clearance enable mobility on uneven terrain. It is equipped with a forward-facing RGB camera (640$\times$480, 30 FPS) for Mask R-CNN-based trail segmentation, a 2D LiDAR sensor with a 12-meter range for obstacle detection, wheel encoders for odometry, and an optional IMU for orientation. These sensors support robust perception and decision-making. Onboard processing is handled by an NVIDIA Jetson Nano running Ubuntu and ROS. A microcontroller manages low-level controls. Power is supplied by a 14.8 V lithium-ion battery, supporting 2 to 4 hours of continuous operation. This configuration enables the seamless integration of Mask R-CNN, SAC, and Pure Pursuit, supporting both the simulation and the real-world deployment of the hybrid navigation model. Figure 3 show the robot used in our experiments.

### Dataset

This dataset is an extended version of the publicly available dataset^52^, further enriched and tailored for robust trail detection in complex forested environments. The dataset used for training our Mask R-CNN model to detect trails in forested environments was gathered in a wilderness setting with the viewpoint height approximating 1.7 meters, similar to that of a human. This height provides an optimal view of the terrain and is a realistic sensor position for ground robots or MAVs, free from obstacles in most cases. The dataset addresses the challenges of wide appearance variability due to factors such as lighting conditions, vegetation types, altitude, and local topography. To capture a comprehensive and diverse set of trail images, we equipped a hiker with three head-mounted cameras: one facing forward and two angled at 30 degrees to the left and right. The cameras, with overlapping fields of view, cover 180 degrees, ensuring robust coverage of the trail environment.

This dataset consists of 8 hours of video footage (1920 × 1080 resolution at 30 fps) from the three GoPro Hero3 Silver cameras, covering 7 km of hiking trails across different altitudes (300–1200 m), times of the day, and weather conditions. The videos were captured during daylight hours to avoid motion blur. Various trail types are represented, from narrow alpine paths to wider forest roads, including difficult or ambiguous sections often encountered in real-world conditions. The dataset contains 23,983 labeled frames (20,072 for training, 2334 for validation, and 1577 for testing), with each frame annotated for trail presence and difficulty level, ensuring the diversity of trail types across both sets. The data split was carefully designed to avoid overlap of trail sections between training and testing, providing a clear evaluation of the model’s performance.

### Network architectures and parameters

The visual perception module was based on the Mask R-CNN architecture with a ResNet-50 backbone and Feature Pyramid Network (FPN) for multi-scale feature extraction. The Region Proposal Network (RPN) was configured to generate 256 proposals per image. The model was implemented in PyTorch using the Detectron2 framework. Training was performed using the Adam optimizer with a learning rate of 0.001 over 40 epochs. The trail segmentation output was post-processed to extract the trail centerline using morphological operations and spline fitting.

The navigation and decision-making component was powered by the SAC algorithm. The SAC agent used two Q-value networks (critics) and one stochastic policy network (actor), each composed of three fully connected layers with 256 hidden units and ReLU activations. The entropy coefficient was automatically tuned during training to balance exploration and exploitation. A discount factor of 0.99 was applied and a replay buffer with a capacity of one million transitions was maintained. The training used mini-batches of 128 transitions sampled from the buffer, and the networks were optimized using the Adam optimizer with a learning rate of $3 \times 10^{-4}$.

### Reward function

In the SAC framework, the agent seeks to learn a stochastic policy $\pi (a_t \mid s_t)$ that maximizes the expected entropy-regularized cumulative return. The optimization objective is defined as

$$J(\pi ) = {\mathbb {E}}_{\pi } \left[ \sum _{t=0}^{\infty } \gamma ^t \left( r(s_t, a_t) + \alpha {\mathscr {H}}(\pi (\cdot \mid s_t)) \right) \right] ,$$

where $\gamma \in (0, 1)$ is the discount factor, $\alpha > 0$ is the temperature coefficient controlling the trade-off between exploration and exploitation, and ${\mathscr {H}}(\pi (\cdot \mid s_t)) = -{\mathbb {E}}_{a_t \sim \pi }[\log \pi (a_t \mid s_t)]$ is the entropy of the policy in the state $s_t$.

To ensure safe, efficient, and goal-oriented navigation, the reward function $r(s_t, a_t)$ was defined as a weighted sum of continuous and discrete behavioral terms:

$$r(s_t, a_t) = w_1 \cdot \phi _{\text {trail}}(s_t) + w_2 \cdot \phi _{\text {progress}}(s_t, a_t) + w_3 \cdot \phi _{\text {goal}}(s_t) - w_4 \cdot \phi _{\text {collision}}(s_t) - w_5 \cdot \phi _{\text {deviation}}(s_t),$$

where $w_i \in {\mathbb {R}}_{\ge 0}$ are manually tuned weights, and the feature terms $\phi _{\cdot }$ are defined as follows:

- *Trail adherence*:
  $$\phi _{\text {trail}}(s_t) = {\mathbb {I}} \left[ {\textbf{x}}_t \in {\mathscr {M}}_{\text {trail}} \right] ,$$
  where ${\mathbb {I}}[\cdot ]$ is the indicator function, ${\textbf{x}}_t \in {\mathbb {R}}^2$ denotes the robot’s position, and ${\mathscr {M}}_{\text {trail}} \subset {\mathbb {R}}^2$ is the segmented trail region.
- *Forward progress*:
  $$\phi _{\text {progress}}(s_t, a_t) = \max (0, v_t \cos (\theta _t - \psi _t)),$$
  where $v_t$ is the linear velocity, $\theta _t$ is the robot’s heading, and $\psi _t$ is the tangent angle of the trail direction.
- *Goal completion*:
  $$\phi _{\text {goal}}(s_t) = {\mathbb {I}} \left[ \Vert {\textbf{x}}_t - {\textbf{x}}_g \Vert _2 < \epsilon \right] ,$$
  where ${\textbf{x}}_g$ is the goal position and $\epsilon$ is the proximity threshold.
- *Collision penalty*:
  $$\phi _{\text {collision}}(s_t) = {\mathbb {I}} \left[ \exists d_i \le d_{\min } \right] ,$$
  where $d_i$ are distance readings from proximity sensors and $d_{\min }$ is a collision threshold.
- *Lateral deviation*:
  $$\phi _{\text {deviation}}(s_t) = \left( \frac{d_{\perp }(s_t)}{d_{\max }} \right) ^2,$$
  where $d_{\perp }(s_t)$ is the perpendicular distance to the trail centerline and $d_{\max }$ is the maximum allowed deviation.

The final reward is both dense and informative, providing gradient-rich learning signals for continuous navigation while embedding discrete safety and success conditions. The weights were empirically set as $w_1 = 1.0$, $w_2 = 0.5$, $w_3 = 5.0$, $w_4 = 1.0$, and $w_5 = 0.75$, with $\alpha$ tuned via automatic entropy adjustment during training.

This reward formulation incentivizes the robot to stay on the trail, avoid collisions, make forward progress, and reach the target, while enabling SAC to efficiently optimize the navigation policy under environmental uncertainty.

### Evaluation metrics

The system was evaluated using standardized perception and navigation metrics to objectively quantify segmentation accuracy and autonomous navigation performance in forest environments.

#### Perception metrics

The visual perception module was evaluated using four widely adopted metrics in the field of image segmentation:

- *Intersection over union (IoU)* measures the overlap between the predicted trail segmentation *P* and the ground truth mask *G*. It is defined as:
  $$\text {IoU} = \frac{|P \cap G|}{|P \cup G|}$$
  where $|P \cap G|$ is the number of pixels common to both the predicted and ground truth masks, and $|P \cup G|$ is the total number of pixels present in either.
- *Pixel-wise accuracy (Acc)* quantifies the proportion of correctly classified pixels across the entire image:
  $$\text {Accuracy} = \frac{TP + TN}{TP + TN + FP + FN}$$
  where *TP* and *TN* represent the number of true positive and true negative pixels, respectively, and *FP* and *FN* denote false positives and false negatives.
- *F1-score* is the harmonic mean of precision and recall, providing a balanced measure of segmentation performance. It is defined as:
  $$\text {F1-Score} = \frac{2 \cdot \text {Precision} \cdot \text {Recall}}{\text {Precision} + \text {Recall}}$$
  with:
  $$\text {Precision} = \frac{TP}{TP + FP}, \quad \text {Recall} = \frac{TP}{TP + FN}$$
- *Dice similarity coefficient (DSC)* evaluates the similarity between the predicted segmentation and the ground truth by measuring their spatial overlap. It is particularly effective in scenarios with class imbalance and is defined as:
  $$\text {DSC} = \frac{2 |P \cap G|}{|P| + |G|}$$
  where |*P*| and |*G*| denote the number of pixels in the predicted and ground truth masks, respectively.

#### Navigation metrics

To assess robot performance in navigating forest trails, we used the following evaluation metrics:

- *Success rate (SR)* is defined as the ratio of successful navigation episodes $N_s$ to the total number of episodes $N_t$, where a success is recorded if the robot reaches the target goal without collision:
  $$\text {Success Rate} = \frac{N_s}{N_t}$$
- *Average time to goal* ($\overline{T}$) measures the mean duration $T_i$ taken by the robot to complete each successful episode:
  $$\overline{T} = \frac{1}{N_s} \sum _{i=1}^{N_s} T_i$$
- *Average lateral deviation* ($\overline{D}$) quantifies the mean perpendicular distance between the robot’s actual path and the centerline of the predicted trail across all steps *t* and episodes $N_t$:
  $$\overline{D} = \frac{1}{\sum _{i=1}^{N_t} T_i} \sum _{i=1}^{N_t} \sum _{t=1}^{T_i} d_{i,t}$$
  where $d_{i,t}$ is the instantaneous lateral deviation at time step *t* in episode *i*.
- *Average number of collisions* ($\overline{C}$) captures the mean number of collisions per episode:
  $$\overline{C} = \frac{1}{N_t} \sum _{i=1}^{N_t} C_i$$
  where $C_i$ is the number of collisions recorded in episode *i*.

All experiments were conducted across three distinct simulated forest environments (Map-A, Map-B, Map-C), with 30 randomized episodes executed in each environment, resulting in a total of 90 trials. The diversity in start and goal positions ensured a robust evaluation across various trail complexities, obstacle distributions, and visual conditions. The reported results represent aggregated metrics averaged over the 90 episodes to ensure statistical significance and mitigate stochastic variability.

## Results analysis

### Trail segmentation

The performance of the Mask R-CNN-based trail segmentation module was quantitatively evaluated on a held-out test set using a combination of overlap-based and pixel-level metrics. The model achieved an IoU of 0.872, a DSC of 0.889, an F1-score of 0.891, and a pixel-wise classification accuracy of 91.2%. Collectively, these results indicate strong agreement between the predicted trail regions and the manually annotated ground truth, while maintaining a balanced trade-off between precision and recall.

The high IoU and DSC values demonstrate that the segmentation output reliably preserves the spatial extent and continuity of the forest trails, which is critical for downstream geometric processing. In particular, accurate segmentation enables stable centerline extraction and curvature estimation, which directly support the state construction of the SAC agent and the curvature-based control commands generated by the Pure Pursuit controller. Importantly, the segmentation accuracy achieved is sufficient to ensure consistent and reliable navigation behavior, as corroborated by the navigation performance and ablation studies presented in subsequent subsections.

In addition to quantitative performance, the segmentation model exhibits strong generalization across visually challenging forest conditions, including partial occlusions caused by vegetation, variations in illumination, cast shadows, and background clutter. The combination of a ResNet-50 backbone with a Feature Pyramid Network (FPN) facilitates robust multi-scale feature extraction, allowing the model to effectively segment trails with varying widths, textures, and visibility levels.

**Fig. 4:**
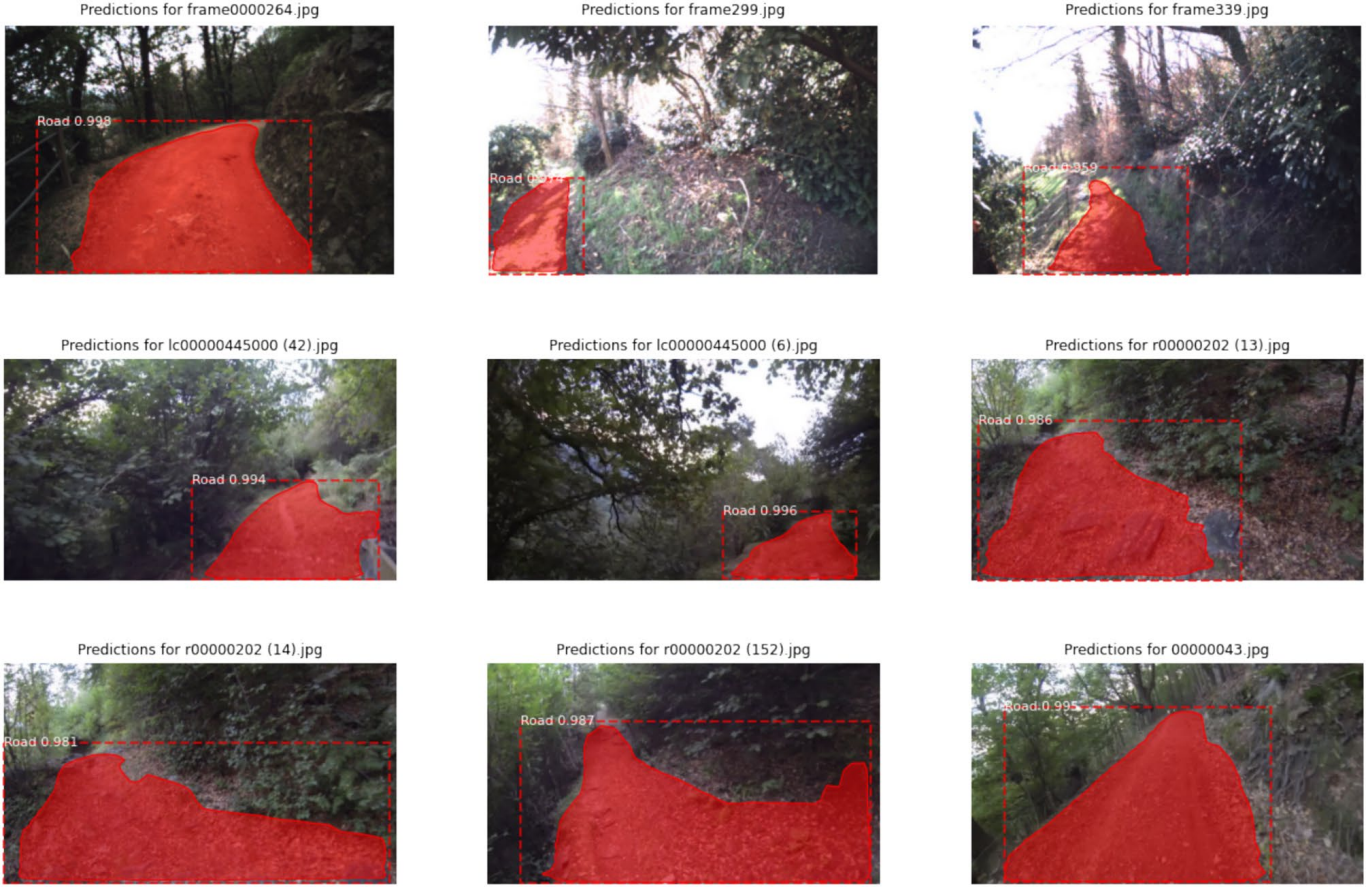
Examples of trail detection results from our visual perception system.

Representative qualitative segmentation results are shown in Fig. 4. These examples illustrate a precise and coherent trail delineation in various environmental scenarios. Even in cases where trail boundaries are poorly defined or partially obscured, the model produces continuous and structurally consistent masks that remain suitable for real-time path extraction. This confirms that the segmentation module fulfills its intended role as a reliable visual perception component rather than as an isolated high-precision segmentation system.

In general, the trail segmentation results demonstrate that the proposed perception module delivers accurate, stable, and computationally efficient visual information. This forms a robust foundation for the subsequent learning-based decision-making and geometric control stages, enabling effective and safe autonomous navigation in complex forest environments.

### Navigation performance

Following the evaluation of the visual perception module, we conducted an extensive series of navigation experiments to assess the integrated system’s performance in dense forest environments. These experiments were designed to test the robot’s ability to follow the predicted trail, avoid obstacles, and reach predefined goal points without human intervention. The hybrid system, composed of Mask R-CNN for trail segmentation, a SAC agent for adaptive policy learning, and a Pure Pursuit controller for smooth trajectory execution, was deployed in three distinct forest maps: Map-A, Map-B, and Map-C, each presenting unique challenges in terms of terrain geometry, vegetation density, and obstacle placement.

Quantitative results show that the system achieved strong performance across all environments. The average success rate across the three maps was 86.7%, meaning that in nearly nine out of ten trials, the robot successfully navigated from its initial position to the designated goal without collisions or getting stuck. This success rate demonstrates the model’s robustness and its ability to generalize across different terrain structures, including sharp turns, narrow passages, and regions with significant foliage coverage or occlusion.

In terms of efficiency, the robot reached the navigation goal in an average time of 21.3 seconds, with relatively low variability across environments (standard deviation of ± 1.1 s). This consistent completion time reflects the effectiveness of the SAC agent in learning time-efficient policies and the contribution of the Pure Pursuit controller in maintaining steady and forward-directed motion.

Another key performance indicator was lateral deviation from the trail centerline, which averaged 0.31 meters across all trials. This metric serves as a proxy for the system’s precision in following the segmented path. The low deviation value highlights the integration’s effectiveness: the Mask R-CNN provided well-centered trail segmentation, and the controller reliably converted these into accurate low-level commands. This is particularly important in forested environments, where narrow trails and limited maneuvering space leave little margin for error.

In addition, the system demonstrated excellent obstacle avoidance behavior. The average number of collisions per episode was only 0.2, indicating that proximity-based feedback and penalization embedded in the SAC reward function successfully discouraged risky maneuvers. The SAC agent learned to steer clear of detected obstacles while still progressing toward the goal, even when faced with occluded or bifurcating paths. These behaviors mirror the complexity of real-world decision-making under uncertainty.

**Fig. 5:**
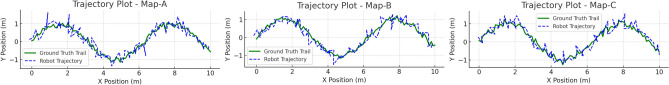
The robot’s trajectories across the three maps. The figure illustrates the paths followed by the robot in three different environments.

Figure 5 presents a visual representation of the actual trajectories of the robot on the three test maps. trajectories adhere well to the predicted trail paths, with particularly good performance in sections that include curves, trail intersections, and visually ambiguous zones. These trajectory plots confirm that the robot not only learned efficient path-following behaviors but also demonstrated reactive adaptability when encountering disturbances or slight misalignments.

The results of the navigation experiments are summarized in Table 1. The breakdown by map further emphasizes the consistency of the system. Although the success rates varied slightly, ranging from 83.3% in Map-C to 90.0% in Map-A, the navigation quality remained high across all metrics. In particular, Map-C, which contained the highest number of visual obstructions and tighter path curvature, still yielded a lateral deviation below 0.35 meters and fewer than 0.3 collisions per run on average. This reflects the system’s capability to maintain safe and reliable operation even in scenarios with limited visibility and constrained maneuverability.

**Table 1:** Navigation performance summary.

Metric	Map-A	Map-B	Map-C	Mean ± Std. Dev
Success rate (%)	90.0	86.7	83.3	86.7 ± 2.7
Avg. time to goal (s)	19.8	22.5	21.7	21.3 ± 1.1
Avg. lateral deviation (m)	0.28	0.34	0.32	0.31 ± 0.03
Avg. collisions per episode	0.1	0.3	0.2	0.2 ± 0.1
Avg. reward per episode	+ 18.3	+ 16.9	+ 17.1	+ 17.4 ± 0.7

These findings confirm the viability of the proposed hybrid model for autonomous navigation in forest environments. The combination of deep perceptual understanding, policy optimization, and geometric control results in a system that is not only accurate and efficient but also resilient to real-time environmental complexity and uncertainty.

### Ablation study

To rigorously evaluate the importance of each core module within the proposed hybrid navigation framework, we conducted a structured ablation study. This experiment was designed to isolate and quantify the impact of the individual components, namely the SAC agent, the Mask R-CNN segmentation model, and the Pure Pursuit controller, by selectively removing or replacing each one with a simpler alternative. The objective was to assess how each module contributes to the system’s overall robustness, precision, and adaptability in dense forest environments.

Three modified configurations were tested in comparison to the original full model. In the first configuration, the SAC reinforcement learning agent was removed and replaced with a rule-based reactive navigation logic. In this setup, decisions regarding steering direction and speed were made using deterministic, hand-coded rules based on proximity sensor readings and the relative position of the trail. Although simple and computationally efficient, this rule-based approach lacks the capacity to learn optimal strategies or adapt to previously unseen conditions.

In the second configuration, the Mask R-CNN perception module was substituted with a traditional computer vision method that relied on color thresholding and basic morphological operations. This approach attempted to detect the trail by identifying dominant color features, such as brown or gray, typically associated with dirt paths. However, such color-based methods are inherently unreliable in complex forest imagery due to environmental factors like variable lighting, shadows, and visually cluttered backgrounds.

In the third configuration, the Pure Pursuit controller was removed and replaced with a naive pose-to-velocity mapping scheme. This method computed motor commands directly from the robot’s relative position to the next waypoint, without accounting for trajectory curvature or applying anticipatory control based on lookahead distance. As a result, the robot lacked the smooth and predictive path-following behavior that is critical for maneuvering through curved and narrow trails.

The outcomes of this study are presented in Table 2. The full model achieved the best overall results, with a success rate of 86.7%, an average of 0.2 collisions per episode, and a trail segmentation Intersection over Union (IoU) of 0.87. These values served as the baseline for evaluating the impact of removing each individual component.

Removing the SAC agent resulted in a noticeable decline in navigation performance. The success rate dropped to 71.1%, and the average number of collisions increased to 0.6 per episode. Although the segmentation IoU remained unchanged at 0.87, due to the continued use of Mask R-CNN, the robot exhibited limited ability to handle ambiguous scenarios such as bifurcating trails or sudden occlusions. This confirms the essential role of SAC in learning adaptive, high-level navigation strategies that cannot be captured through static, rule-based logic.

The most severe degradation occurred in the configuration without the Mask R-CNN module. Replacing it with a basic color segmentation method significantly reduced the IoU to 0.48, indicating poor segmentation accuracy. This substantial drop in perception quality had a cascading effect on navigation. The success rate fell to 63.4%, and the robot experienced an average of 1.1 collisions per episode. These results confirm that robust trail perception is fundamental for ensuring safe and accurate navigation, particularly in environments where visual ambiguity is common.

In the final configuration, the removal of the Pure Pursuit controller led to erratic and unstable robot movement. Although both SAC and Mask R-CNN remained intact and continued to provide accurate decision-making and trail segmentation, the lack of geometric path-following control resulted in imprecise execution of navigation commands. The success rate in this configuration decreased to 74.5%, and the collision rate rose to 0.5 per episode. Although the IoU of segmentation remained high at 0.87, the robot’s movements lacked the smoothness and anticipatory adjustments required for efficient trail tracking, especially in curved segments.

Overall, the results of the ablation study clearly demonstrate that the success of the proposed system depends on the integration of all three components. Each module contributes a distinct and essential function: Mask R-CNN provides accurate and reliable trail segmentation, SAC enables adaptive and strategic decision-making in complex conditions, and the Pure Pursuit controller ensures precise and stable execution of navigation trajectories. The degradation observed when any component is removed validates the design choices made in constructing the entire system and underscores the importance of holistic integration for robust autonomous navigation in natural environments.

**Table 2:** Ablation study—Component impact on navigation.

Configuration	Success rate (%)	Avg. collisions	IoU (trail)
Full model (ours)	86.7	0.2	0.87
Without SAC (rule-based)	71.1	0.6	0.87
Without mask R-CNN (color seg.)	63.4	1.1	0.48
Without pure pursuit	74.5	0.5	0.87

### Learning progress of the SAC agent

The learning dynamics of the proposed Soft SAC agent was analyzed by tracking the average episodic reward throughout training, which serves as a quantitative indicator of policy improvement and behavioral stabilization. Training was carried out for approximately $1.5 \times 10^{5}$ environment interaction steps, during which the agent exhibited a clear and progressive increase in cumulative reward, reflecting effective policy optimization and stable convergence.

During the initial phase of training, the reward signal exhibited high variance and relatively low mean values. This behavior is characteristic of entropy-regularized reinforcement learning methods, in which the policy deliberately prioritizes exploration and frequently samples suboptimal actions. At this stage, the agent actively explores alternative navigation strategies, often deviating from the trail or executing inefficient maneuvers, to learn the structure of the environment and the consequences of its control decisions.

As training progresses, the variance in episodic returns gradually decreases while the mean reward consistently increases, indicating a transition from exploration-dominated behavior toward exploitation of learned navigation strategies. The agent increasingly favors smooth trail-following trajectories, maintains safer distances from obstacles, and reduces unnecessary heading corrections. These improvements are directly driven by the reward design, which explicitly promotes forward progress along the segmented trail while penalizing collisions, excessive curvature, and unstable motion.

**Fig. 6:**
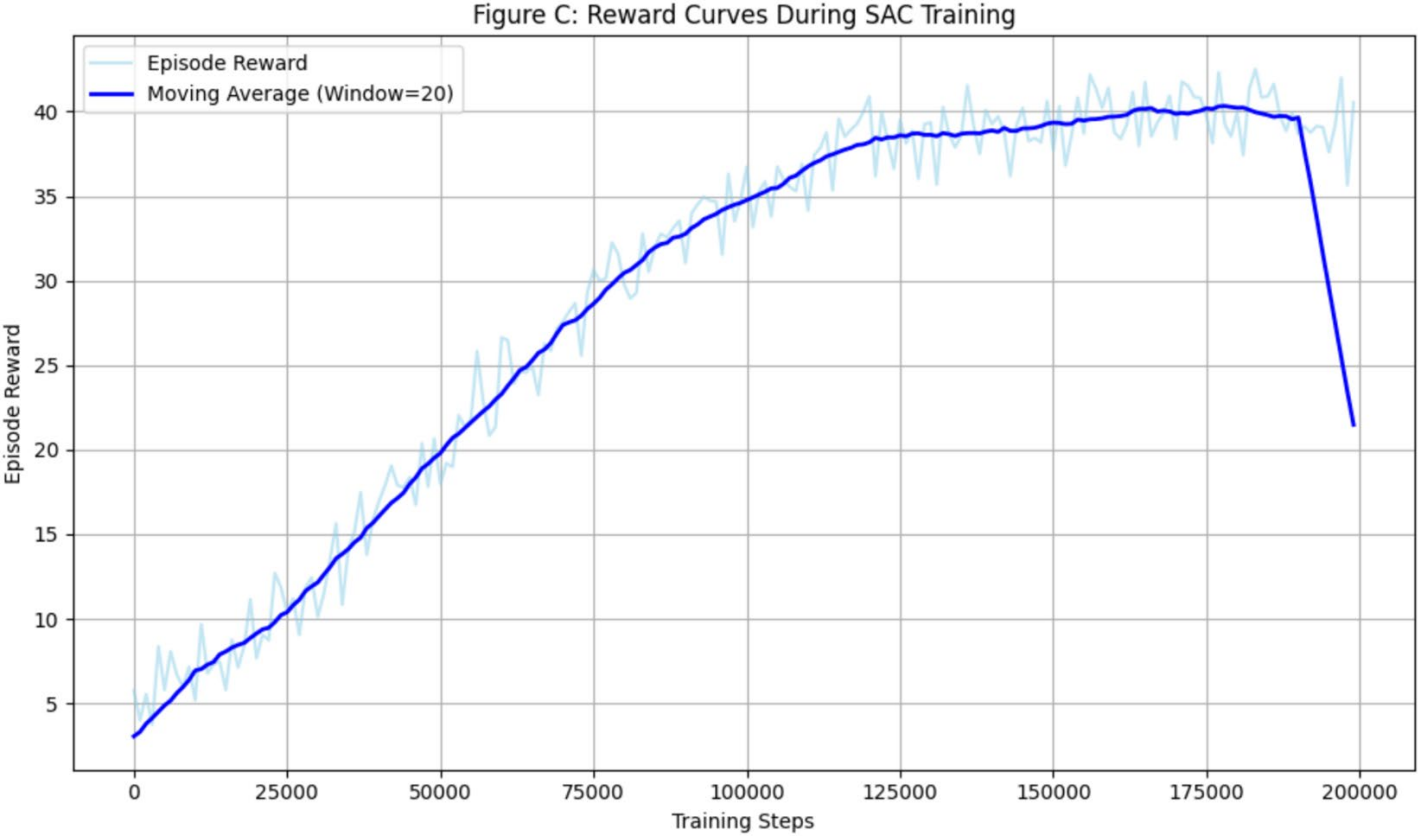
Evolution of the average episodic reward during SAC training. The moving average highlights the progressive improvement and convergence of the learned navigation policy.

Figure 6 illustrates the evolution of episodic reward during training. To emphasize long-term learning trends, a moving average filter is applied to the raw reward signal. A transient deviation between the moving average and the underlying episodic rewards can be observed during the intermediate training stages. This temporary drop does not indicate instability of the learning process; rather, it arises from the combined effects of entropy-driven exploration, environmental stochasticity, and occasional episodic failures. Even after the policy begins to improve, SAC maintains exploratory behavior through entropy maximization, which can sporadically result in suboptimal action sequences, premature episode termination, or collisions. When several such low-reward episodes occur within a short temporal window, their cumulative influence is amplified by the moving average, producing a noticeable downward deviation in the smoothed curve despite the presence of higher-reward episodes in the raw signal.

Furthermore, the partially observable and unstructured nature of the forest environment introduces additional variability through segmentation noise, appearance changes along the trail, and minor obstacle perturbations, which can momentarily degrade performance. Importantly, the deviation remains localized and is followed by a rapid recovery of the moving average, confirming that the learned policy remains stable and resilient.

To quantitatively assess convergence, we define the policy as converged when the slope of the moving average reward falls below a small threshold $\epsilon$ and its variance remains bounded over a fixed evaluation window. Formally, convergence is achieved at time step $t_c$ if:

$$\left| \frac{1}{K} \sum _{k=0}^{K-1} \left( \bar{R}_{t_c-k} - \bar{R}_{t_c-k-1} \right) \right|< \epsilon \quad \text {and} \quad \textrm{Var}\left( \{ \bar{R}_{t_c-K}, \dots , \bar{R}_{t_c} \} \right) < \sigma ^2,$$

where $\bar{R}_t$ denotes the moving average reward at step *t*, *K* is the window length, and $\epsilon$ and $\sigma ^2$ are empirically selected thresholds. Under this criterion, the SAC agent consistently satisfies convergence conditions in the later stages of training, as evidenced by the observed reward plateau.

The eventual stabilization of the reward curve indicates convergence to a consistent performance regime, demonstrating that the SAC agent acquires a robust navigation strategy suitable for deployment in complex, obstacle-rich forest environments within a practical training horizon.

### Comparative performance analysis

To rigorously evaluate the efficacy and generalizability of the proposed vision-based hybrid navigation framework, an extensive comparative performance analysis was conducted against several state-of-the-art and contemporary methods spanning multiple perception paradigms: classical machine learning, feature-based navigation, end-to-end deep learning, memory-augmented reinforcement learning, and multimodal sensor-fusion approaches. The selected baselines encompass vision-only and LiDAR-inclusive systems to provide a holistic assessment of perceptual-modality trade-offs in unstructured forest environments. Specifically, we compare against:^21^, which employs a classical image classification pipeline for trail detection;^24^, a lightweight handcrafted-feature approach for forest navigation;^25^, an end-to-end learned vision-based navigation model;^46^, a semantic memory-augmented deep RL method originally designed for indoor settings;^53^, a forestry robot that integrate vision and LiDAR for navigation; and^54^, a purely LiDAR-based traversability estimation framework.

All methods were evaluated in the same three simulated forest environments (Map-A, Map-B, Map-C) using navigation metrics. Quantitative results are summarized in two tables: Table 3 details per-environment success rates across the three simulated forest maps, while Table 4 presents aggregated navigation metrics including average success rate, completion time, lateral deviation, and collision frequency. The proposed system consistently outperformed all vision-only baselines in all performance indicators. The classical method of^21^ exhibited limited spatial precision, attributed to its reliance on image-level classification, which fails to capture fine-grained trail geometry. The lightweight feature-based approach of^24^ demonstrated computational efficiency, but suffered significant performance degradation under partial occlusions and variable illumination. In contrast, the end-to-end model of^25^ achieved a stronger overall performance through learned feature representations, but still underperformed relative to our framework in terms of success rate and collision avoidance. The indoor-focused method by^46^, while effective in structured settings, struggled to generalize to outdoor terrain variability and exhibited suboptimal trail continuity reasoning.

**Table 3:** Success rates (%) across different forest maps.

Method	Sensor modality	Map-A	Map-B	Map-C
^21^	Vision (RGB)	73.3	68.3	70.0
^46^	Vision (RGB)	85.0	78.3	80.0
^24^	Vision (RGB)	80.0	75.0	76.7
^25^	Vision (RGB)	83.3	80.0	81.7
^53^	Vision + LiDAR	81.7	**88.3**	76.7
^54^	LiDAR-only	83.3	86.7	78.3
Our	Vision (RGB) Only	**90.0**	86.7	**83.3**

Bold numbers represent the highest success rates (%) for each forest map. For Maps A and C (columns 3 and 5), our proposed method achieved the best success rate. For Map B (column 4), the method cited in [53] (row 6) achieved the best success rate. The last column shows the average success rate across the three maps, with the best value highlighted in bold.

The multimodal and LiDAR-based approaches demonstrated clear modality-dependent strengths. In Map-B, where accurate 3D geometry perception is paramount, both the vision–LiDAR fusion system^53^ and the LiDAR-only method^54^ achieved high success rates. By contrast, their performance substantially declined in Map-C, a scenario in which semantic disambiguation of trail identity supersedes geometric traversability. This underscores a key limitation of purely geometric sensing: while resilient to visual noise and lighting variation, LiDAR cannot deduce navigational intent or differentiate between semantically distinct but geometrically similar paths.

**Table 4:** Aggregated navigation performance (average across all maps).

Method	Avg. success rate (%)	Avg. time to goal (s)	Avg. lateral deviation (m)	Avg. collisions/episode
^21^	70.5	29.8	0.55	1.1
^46^	81.1	24.1	0.40	0.6
^24^	77.2	26.3	0.48	0.8
^25^	81.7	23.9	0.39	0.5
^53^	82.2	22.8	0.35	0.4
^54^	82.8	22.5	0.34	0.4
Our	**86.7**	**21.3**	**0.31**	**0.2**

Bold numbers represent the best values for each metric (success rate, completion time, lateral deviation, and collision frequency) averaged across all maps.

Our framework achieved the highest overall success rate (86.7%) and the lowest collision frequency (0.2 per episode) among all the evaluated methods. Notably, it also exceeded both multimodal and LiDAR-only baselines in aggregate navigation performance, demonstrating that a well-designed vision-only system can rival or exceed the robustness of geometric sensing in semantically complex unstructured environments. This result highlights the complementary roles of deep visual perception, which provides rich contextual and directional cues, and reinforcement learning, which enables adaptive decision-making under perceptual uncertainty.

The comparative analysis further elucidates inherent trade-offs between perceptual modalities. LiDAR-based systems excel in geometrically complex and visually degraded conditions but lack semantic scene understanding. Vision-only systems offer superior semantic interpretability and lower sensor cost but are susceptible to photometric variations and occlusions. Our framework mitigates these limitations through a synergistic integration of learning-based perception and control, enabling robust trail following without reliance on external localization or prior maps.

This comparative study validates that the proposed vision-only hybrid navigation system establishes a new state-of-the-art for autonomous forest trail following, outperforming a diverse set of recent vision-based, LiDAR-based, and multimodal methods across multiple metrics of navigation efficacy. The results affirm the viability of combining deep semantic segmentation with entropy-regularized reinforcement learning and classical control for robust, cost-effective, and deployable autonomous navigation in GPS-denied natural environments.

## Discussion

The proposed navigation system demonstrated robust and reliable performance across a range of simulated dense forest environments. Quantitative results from both navigation experiments and comparative analysis confirm the system’s strengths in perception accuracy, motion stability, and policy adaptability, while also revealing several areas where further refinement is warranted.

### Training dynamics

Throughout training, the SAC agent exhibited a clear policy improvement, as indicated by the monotonic increase in the average episodic reward over approximately $1.5 \times 10^5$ interaction steps, as shown in Fig. 6. The learning curve followed a characteristic pattern: an initial phase of high variance due to exploratory actions, followed by a steady increase in reward as the agent learned to associate trail-aligned motion with positive returns. Notably, the entropy regularization term played a dual role: early in training, it encouraged broad exploration of the action space, while later it helped prevent premature convergence to suboptimal deterministic policies. The reward reached a plateau after approximately 120,000 steps, indicating that the agent converged to a near-optimal policy for the given environment and reward shaping. This convergence behavior aligns with established SAC benchmarks for continuous control tasks. However, the partially observable nature of forest navigation introduced additional variance not typically seen in fully observable simulated domains.

### Emergent navigation behaviors

Beyond quantitative metrics, several emergent qualitative behaviors underscored the system’s adaptability. The robot successfully navigated partially occluded trails by executing short-term detours when obstructions were encountered, a capability attributed to the SAC’s entropy-augmented objective, which encourages cautious exploration in uncertain states. This was particularly evident in Map-A, where overhanging vegetation frequently obscured the trail centerline. Rather than stopping or diverging completely, the agent learned to rely on proximal trail segments and extrapolate likely path continuity. This represents a form of short-horizon inference that was not explicitly encoded in the reward function.

Furthermore, the integration of the Pure Pursuit controller ensured smooth and kinematically consistent motion, particularly in curved trail segments where reactive control methods often exhibit oscillatory behavior. The blending mechanism between SAC actions and Pure Pursuit commands allowed the system to balance adaptability and stability: in straight, well-defined trail sections, Pure Pursuit dominated, ensuring efficient progress; in ambiguous or cluttered regions, SAC exerted greater influence, enabling obstacle avoidance and recovery behaviors. This hybrid control strategy proved critical in Map-C, where false trails and bifurcations required semantic discrimination rather than purely geometric path following.

The system also demonstrated an ability to tolerate transient segmentation errors: rather than overreacting to noisy perceptual inputs, the policy often delayed major steering adjustments until visual evidence was reaffirmed, mirroring conservative human driving strategies and enhancing operational safety. This behavior emerged despite the absence of explicit filtering or temporal smoothing in the perception pipeline, suggesting that the SAC policy learned to implicitly account for perceptual uncertainty through its state representation and value function.

The comparative analysis further contextualizes these findings. Although the proposed system outperformed both vision-only and LiDAR-based baselines, the performance gap narrowed in geometrically complex terrain (Map-B), where LiDAR methods excelled. This suggests that future versions of the system could benefit from optional LiDAR fusion in environments where geometric precision is paramount. This could be implemented either through the late fusion of traversability estimates or the early fusion of multimodal features. Conversely, in semantically complex settings (Map-C), the vision-only approach proved superior, underscoring the importance of semantic scene understanding for trail disambiguation. This duality highlights a fundamental trade-off in outdoor robot perception. Although geometric sensing provides metric accuracy, it lacks semantic grounding; visual perception, on the other hand, offers rich semantics but is metrically ambiguous and environmentally sensitive.

In particular, the proposed system achieved its leading performance without expensive 3D sensors or detailed prior maps, positioning it as a cost-effective and scalable solution for field robotics. Its modular architecture facilitates incremental improvements and aligns with recent trends toward hybrid navigation systems that blend data-driven and model-based approaches. This work contributes to the growing research on learning-based navigation in unstructured environments by demonstrating that a structured hybrid system can achieve robust autonomy without end-to-end black-box training. Specifically, it effectively combines deep learning for perception, reinforcement learning for decision-making, and classical control for execution. The interpretability of each module also simplifies debugging and safety analysis, which is an important consideration for real-world deployment.

### Limitations

Despite these strengths, several limitations were identified through both ablation studies and comparative evaluation.

#### Perceptual sensitivity

The perception module remains sensitive to extreme illumination variations, such as high-contrast shadows, sun glare, or low-angle lighting. These conditions can degrade Mask R-CNN segmentation quality and occasionally induce navigation errors. Although the model was trained on a diverse dataset including various times of day and weather conditions, certain challenging photometric conditions, like dappled sunlight through dense canopy, were underrepresented. This led to occasional segmentation failures, which typically manifested as fragmented trail masks or false positive detections in shaded regions.

Although the SAC policy exhibited some capacity to recover from such perceptual disturbances, often by slowing down and relying more heavily on proximity sensors, a systematic improvement of visual robustness remains a clear direction for future work. Potential techniques to achieve this include domain-invariant representation learning, multi-spectral imaging, and test-time augmentation.

#### Exploration–exploitation trade-off

In visually ambiguous or open terrain, the SAC agent’s entropy-maximizing objective occasionally led to excessive exploration. This resulted in suboptimal path choices and increased navigation time. The behavior was most apparent in Map?C near trail intersections, where the agent sometimes ventured onto false paths before correcting its course. While beneficial during training for discovering robust policies, such exploration may hinder deployment efficiency.

Adaptive entropy tuning could mitigate this issue without sacrificing exploratory capacity during learning. For example, annealing the temperature parameter $\alpha$ based on episode progress or uncertainty estimates would help focus the agent over time. Alternatively, introducing a notion of “commitment” to chosen paths, through action persistence or hysteresis, could reduce dithering in ambiguous zones.

#### Memoryless policy limitation

A third limitation stems from the system’s lack of explicit temporal memory. Because the policy operates on a Markovian state representation, performance degrades when the trail is occluded over multiple consecutive frames. In such scenarios, the robot exhibited hesitation or erratic motion due to an inability to infer trail continuity from historical context. This was observed in Map-B where fallen logs created extended visual obstructions. Incorporating recurrent neural networks or transformer-based sequence models into the state encoder could enable temporally coherent reasoning and improve resilience to intermittent perceptual dropout. Moreover, explicit memory of past actions and observations could help the agent maintain a consistent heading during temporary blindness, a capability crucial for real-world forest navigation.

#### Generalization beyond training distribution

While the system generalized well across the three designed maps, its performance on truly novel trail types (e.g., snow-covered paths, or muddy terrain) remains untested. The current perception module was trained exclusively on forest trail imagery from a specific geographic region; expanding the training dataset to include broader environmental diversity would likely improve cross-domain robustness. Additionally, the simulation-to-reality gap presents a significant challenge: although the simulator included realistic vegetation and lighting models, real-world factors such as dust, lens flare, and dynamic obstacles (e.g., moving animals) were not modeled.

#### Integration with real-world deployment

The framework aligns with recent trends toward simulation-trained policies for field robotics. By leveraging high-fidelity simulated environments, we were able to conduct extensive ablation studies and comparative evaluations that would be impractical in physical forests. However, the observed limitations underscore the need for better sim-to-real transfer techniques, particularly for visual perception where domain gaps are pronounced. Future work should also explore extensions to multi-robot systems, long-term autonomy with lifelong learning, and integration with higher-level task planning for applications in environmental monitoring, search-and-rescue, and precision forestry.

## Conclusion

This paper presents a hybrid autonomous navigation system designed for robust robot mobility in dense, unstructured forest environments. The proposed architecture integrates Mask R-CNN for pixel-accurate semantic trail segmentation, the SAC algorithm for adaptive decision-making under continuous action spaces, and a Pure Pursuit controller for stable and smooth path tracking. By combining deep visual perception with entropy-regularized reinforcement learning and geometric control, the system operates without reliance on GPS or prior maps, which is a critical advantage in real-world forest settings.

Through extensive experimentation in high-fidelity simulated forest environments featuring diverse terrain geometries, vegetation densities, and visual complexities, the system demonstrated strong performance across multiple quantitative metrics: an average trail-following success rate of 86.7%, low lateral deviation (0.31 m), minimal collisions (0.2 per episode) and consistent goal-reaching efficiency (21.3 s average). Ablation studies confirmed the indispensable role of each module: Mask R-CNN provided reliable trail perception even under occlusion, SAC enabled adaptive and safe decision-making in ambiguous regions, and Pure Pursuit ensured kinematic feasibility and motion smoothness. The comparative analysis further established that the proposed vision-only hybrid framework outperforms recent LiDAR-based, feature-based, and end-to-end learning alternatives, particularly in semantically complex trail networks where appearance cues outweigh geometric traversability.

A key contribution of this work is its modular and interpretable design, which decouples perception, decision-making, and control into functionally transparent subsystems. Unlike monolithic end-to-end models, this structure supports incremental improvement, safety analysis, and transfer across domains while maintaining real-time performance on embedded hardware. The integration of pixel-level semantic segmentation with a continuous control RL agent also represents a meaningful step toward semantically grounded navigation in visually cluttered outdoor environments.

Despite these promising results, several limitations were identified that point toward future research directions. First, the system’s perception module remains sensitive to extreme illumination variations, suggesting the need for robustness enhancements through domain adaptation, multi-spectral sensing, or invariant feature learning. Second, the lack of explicit temporal memory limits performance during extended visual occlusions; incorporating recurrent or attention-based state encoders could improve trail continuity reasoning. Third, while simulation experiments provide a controlled testbed, real-world deployment will require addressing the sim-to-real gap through targeted fine-tuning, sensor fusion, and validation in physical forest settings.

Beyond single-robot navigation, this framework provides a foundation for several impactful extensions. These include multi-agent coordination for distributed environmental monitoring, integration with aerial or ground sensor networks, lifelong adaptation to seasonal and ecological changes, and application to related domains such as agricultural robotics, search-and-rescue operations, and planetary exploration. The modularity of the system also facilitates the incorporation of additional sensing modalities, such as LiDAR, for enhanced geometric perception, without requiring a full architectural redesign.

This work demonstrates that hybrid systems that combine deep visual perception, reinforcement learning, and classical control can achieve robust, efficient, and safe autonomous navigation in challenging natural environments. By balancing data-driven adaptability with model-based stability, the proposed approach offers a scalable and practical pathway to the deployment of autonomous robots in real-world forestry and field robotics applications.

## Data Availability

The data presented in this study are available on request from the corresponding author. The data is publicly available.
